# Ginsenoside Rh2 Ameliorates Doxorubicin-Induced Senescence Bystander Effect in Breast Carcinoma Cell MDA-MB-231 and Normal Epithelial Cell MCF-10A

**DOI:** 10.3390/ijms20051244

**Published:** 2019-03-12

**Authors:** Jin-Gang Hou, Byeong-Min Jeon, Yee-Jin Yun, Chang-Hao Cui, Sun-Chang Kim

**Affiliations:** 1 Intelligent Synthetic Biology Center, Daejeon 34141, Korea; houjg2014@kaist.ac.kr (J.-G.H.); chcui@kaist.ac.kr (C.-H.C.); 2 Department of Biological Sciences, KAIST, Daejeon 34141, Korea; jbm0901@kaist.ac.kr (B.-M.J.); yyj07@kaist.ac.kr (Y.-J.Y.)

**Keywords:** cellular senescence, doxorubicin, breast cancer cell, breast epithelial cell, ginsenoside Rh2

## Abstract

The anthracycline antibiotic doxorubicin is commonly used antineoplastic drug in breast cancer treatment. Like most chemotherapy, doxorubicin does not selectively target tumorigenic cells with high proliferation rate and often causes serve side effects. In the present study, we demonstrated the cellular senescence and senescence associated secretory phenotype (SASP) of both breast tumor cell MDA-MB-231 and normal epithelial cell MCF-10A induced by clinical dose of doxorubicin (100 nM). Senescence was confirmed by flattened morphology, increased level of beta galactose, accumulating contents of lysosome and mitochondrial, and elevated expression of p16 and p21 proteins. Similarly, SASP was identified by highly secreted proteins IL-6, IL-8, GRO, GM-CSF, MCP-1, and MMP1 by antibody array assay. Reciprocal experiments, determined by cell proliferation and apoptosis assays and cell migration and cell invasion, indicated that SASP of MDA-MB-231 cell induces growth arrest of MCF-10A, whereas SASP of MCF-10A significantly stimulates the proliferation of MDA-MB-231. Interestingly, SASP from both cells powerfully promotes the cell migration and cell invasion of MDA-MB-231 cells. Treatment with the natural product ginsenoside Rh2 does not prevent cellular senescence or exert senolytic. However, SASP from senescent cells treated with Rh2 greatly attenuated the above-mentioned bystander effect. Altogether, Rh2 is a potential candidate to ameliorate this unwanted chemotherapy-induced senescence bystander effect.

## 1. Introduction

Chemotherapeutic drugs are designed to eliminate tumor cells with high proliferation rates in treatment of malignancies [1]. Generally, tumor cells are deprived of reproductive potential and undergo apoptosis [2]. However, there is a growing body of literature that recognizes apoptosis may not be the confined mechanism whereby cancer cells lose their ability of self-renewal after exposure to chemotherapy treatment, especially in solid tumors [3]. Among these alternative pathways, the theory of cellular senescence provides a useful account of how thermotherapy prevents tumor growth both in vitro and in vivo [4]. Cellular senescence is characterized by an irreversible cellular growth arrest in response to DNA damage. Additionally, cellular senescence was proposed to be regulated by two main tumor suppressor pathways of cell, the ARF/53 and INK4a/RB pathways [5]. Since most clinical or under-investigation chemotherapeutic drugs induce severe DNA damage to tumor cells, they are conceivably engaged and, correspondingly, trigger cellular senescence. However, the tumor suppression of senescence is quickly challenged by massive emerging envidence. Senescent cells still metabolically active preserving the potential to secrete paracrine acting factors [6]. Manipulation of p53 and p16 activation can reverse cellular senescence in human cells, suggesting that chemotherapy resistance may be partly driven by the subsets of tumor cells emerging from a senescence state [7]. In addition, DNA damage-induced cellular senescence develops a typical senescence-associated secretory phenotype (SASP), involving a spectrum of secreted growth factors, proteases, and cytokines, many of which are attributive to the microenvironment promoting resistance [8]. On the other hand, most cytotoxic chemotherapy agents target cancer cell by sensing tumor cell characteristics, such as high proliferation rates. However, this manner also targets normal counterparts and senescent cell disrupts the normal function of tissues and organs [9]. Evidence suggests that a regimen with anthracycline and alkylating agents on patients with breast cancer durably induces cellular senescence and SASP [10]. Interestingly, senescent cells, especially SASP, are often recognized and cleared through an antigen-specific immune response termed senescence surveillance [11], which might be a potential strategy to treat cancer patients. However, SASP also can promote the proliferation and invasion of neighboring cancer cells that escape the senolytic and immune system, leading to cancer relapse [12]. Importantly, the accumulation of senescent cells in tissue with age gives rise to chronic maintenance of senescent cells since the immune system declines in function during aging [13]. This indicates that targeting senescent cells and SASP could be a strategy for cancer therapy and aging-related cancer relapse.

Ginsenoside is of interest because it has been receiving considerable attention as a general tonic on longevity and body enhancement [14,15]. Recently, there has been renewed interest in synergistic effects of ginsenoside on chemotherapy treatment. Ginsenoside Rh2 treatment combined with selected chemotherapy agents sensitizes toxicity on prostate cancer cells [16] and hepatoma cells [17], and inhibits angiogenesis and growth of lung cancer [18] and ovarian cancer [19]. Rh2 enhances efficacy of paclitaxel or mitoxantrone in prostate cancer cells [20]. Accordingly, these above synergistic effects are mainly due to regulation of drug efflux, enhancement of subcellular distribution, and induction of apoptosis. Importantly, the interaction between ginsensoides and chemotherapy agents allows lower doses in clinical application. Furthermore, this chemotherapy regimen is reported to induce unwanted impairment and a proposed protection of ginsenoside Rh2 reverses this side effect in mice with lung tumor [21]. Collectively, ginsenoside Rh2 possesses great potential in sensing chemotherapy agents and ameliorating unwanted side effects.

In the present study, we determined the effects of ginsenoside Rh2 on the following circumstances. (1) Senolytic effect on Doxorubicin-induced cellular senescence of breast cells. (2) Doxorubicin induces cellular senescence of breast epithelial cells. (3) Senescent breast cancer cells induces bystander effects on normal counterparts. (4) Senescent normal counterparts stimulate proliferation, migration, and invasion of breast cancer cells. The present work suggested that Rh2 extenuated the bystander effect induced by chemotherapy in breast cells.

## 2. Results

### 2.1. Low Dose Doxorubicin Induces Senescence of Human Breast Cells

**Figure 1:**
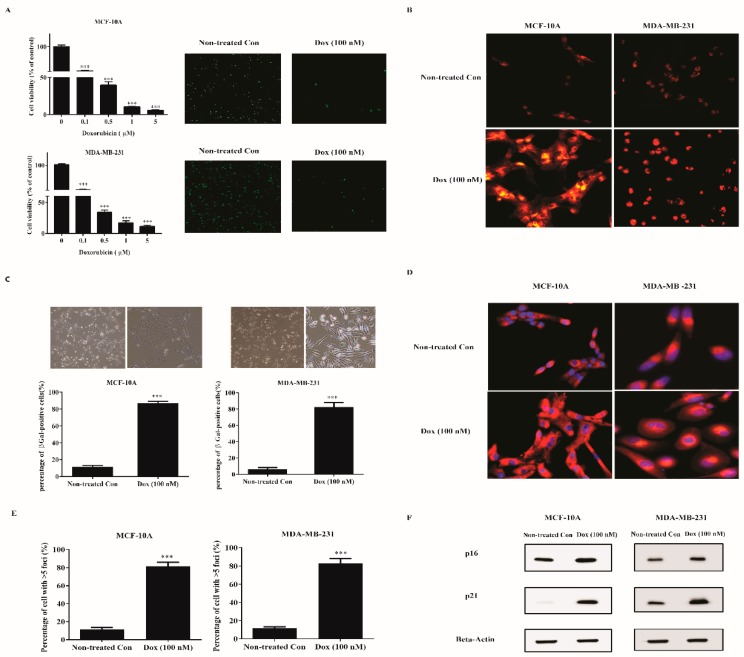
Doxorubicin induces senescence of breast normal and cancer cells. (**A**) MDA-MB-231 cells and MCF-10A cells were treated with indicated doses of doxorubicin for 72 h. Cells were either assayed for viability (left panel) or fixed and stained for Ki-67 (right panel, green). Cell viability was determined by WST-1 method and normalized with nontreated con cells. (**B**–**D**) Cells were treated with 100 nM doxorubicin for 72 h to induce senescence. Then, cells (**B**) were incubated with Lysotracker Red (200 nM) for 1 h. Cells (**C**) were fixed and stained for SA-β-gal. The upper panel shows the bright-field images. The lower panel was the percentage of positive cells (>200 cells scored). Cells (**D**) were incubated with Mitotracker Red (100 nM) for 30 min. Blue, DAPI stained nuclear. Representative images were captured by a Fluorescence Microscope (100× for **A**,**C**; 200× for (**B**,**D**). (**E**) Then days after senescence induction, nontreated con and senescent cells were immunostained with 53BP1, a DNA-SCAR marker. The number of the foci was determined by CellProfiler. Shown was the percentage of cells with >5 foci. (**F**) Extracts from nontreated con and senescent cells were measured for the indicated proteins by western blotting. Beta-actin was used as the loading control. *** indicates *p* < 0.001 versus nontreated con.

To screen out the concentration of doxorubicin to induce senescence in human breast cells, MDA-MB-231, and MCF-10A cells were exposed to various concentrations of doxorubicin for 72 h and cell viability was determined by WST-1 method. Doxorubicin concentration-dependently reduced the cell viability of both cell lines (Figure 1A). We observed that concentrations initiating from 0.1 to 5 μM significantly decreased the cell viability than vehicle (distilled water) control. However, concentrations higher than 0.1 μM induced evidenced cellular apoptosis with a large amount of debris. Hence we used 0.1 μM doxorubicin for subsequent experiments. Concomitantly, immunostaining for Ki-67, a proliferation marker, corroborated the profound cell growth arrest at 100 nM doxorubicin.

To further identify whether cells with inhibited growth turned senescent, we evaluated typical markers for senescence. One biomarker of senescence is the accumulating lysosomal contents. Non-treated and treated (100 nM doxorubicin) cells were labeled with Lysotracker Red (Figure 1B). Notably, treated cells displayed a marked redistribution of lysosome with diffused perinuclear pattern. Apart from enhanced lysosomal content, an increased percentage of canonical marker SA-β-gal in treated cells was correspondingly observed (Figure 1C). Another biomarker is increased mitochondrial biomass. We therefore labeled the non-treated and treated (100 nM doxorubicin) cells with Mitotracker Red (Figure 1D). A remarkable mitochondrial signal was detected in treated cells. Senescent cells showed nuclear foci termed DNA-SCARs, requiring for SASP development. Treated cells significantly altered the number of 53BP1 foci compared with Nontreated con (Figure 1E). Senescence was further confirmed by elevated levels of proteins p16 and p21 in treated cells using Western blot analysis (Figure 1F). Importantly, the above evaluations indicated that 100 nM doxorubicin induces typical cellular senescence in human breast cell lines.

### 2.2. Doxorubicin-Induced SASP in Human Breast Cell Lines

**Figure 2:**
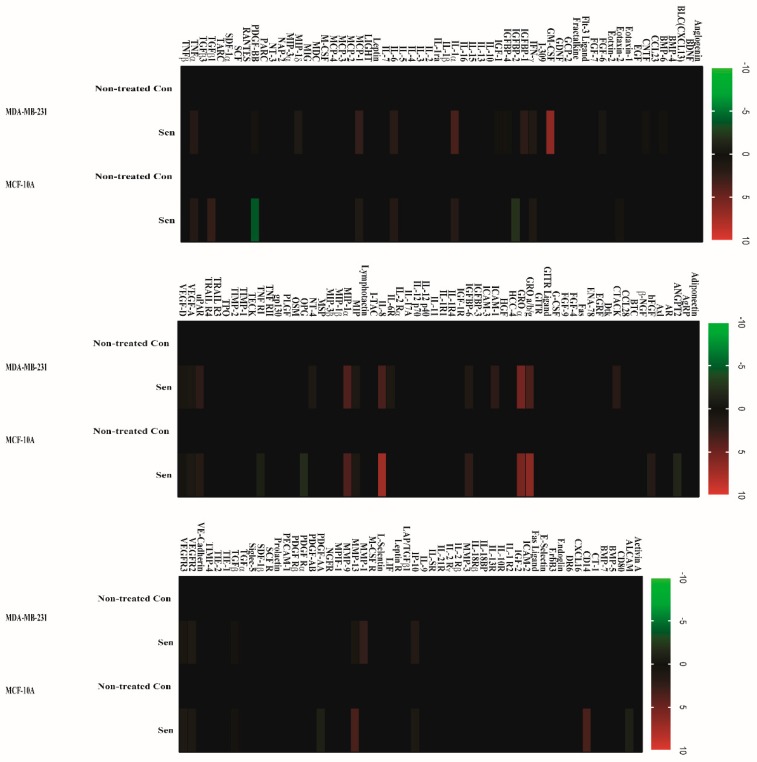
Senescent human breast cancer and normal cells developed SASP. Conditioned medium from nonsenescent (nontreated Con) or senescent (100 nM of doxorubicin exposure, Sen) MDA-MB-231 (A) and MCF-10A (B) cells were analyzed with human cytokine antibody arrays. Levels of each cytokine factor in untreated cells were arbitrary set to zero. Data shown represent log2-fold change in expression relative to untreated cells. Signals higher than the untreated control are shown in red; signals lower than the untreated control are shown in green.

To determine whether senescent cells developed SASP, a conditioned medium from senescent MDA-MB-231 and MCF-10A cells was applied to a human cytokine array assay with 120 secreted proteins. In contrast to nontreated con cells, for senescent human breast cancer MDA-MB-231 cells, the factors detected by arrays and secreted at a significant level are FGF-6, GM-CSF, IGFBP-1, MCP-1, IL-6, IL-1α, GRO a/b/g, GRO α, IL-8, MIP, MIP-1α, uPAR, ICAM-1, and MMP-1(Figure 2). In senescent nontumorigenic MCF-10A cells, proteins secreted at substantial level are FGF-6, MCP-1, GRO a/b/g, GRO α, IL-8, uPAR, IGFBP-6, OPG, TNFR1, IP10, CD14, and MMP-13 (Figure 2). Additionally, we observed in certain proteins (PDGF-AA, PDGF-BB, ANGPT2, IGFBP-2, and ALCAM) that secretion was downregulated in senescent MCF-10A cells. Intriguingly, although a similar secretion pattern of major SASP factors such IL-6 and IL-8 was observed in both cell lines, two cell lines displayed differed secretory phenotype. We postulated that these differences may lead to various paracrine effects.

### 2.3. SASP Stimulates Migration and Invasion of Breast Cancer Cells

**Figure 3:**
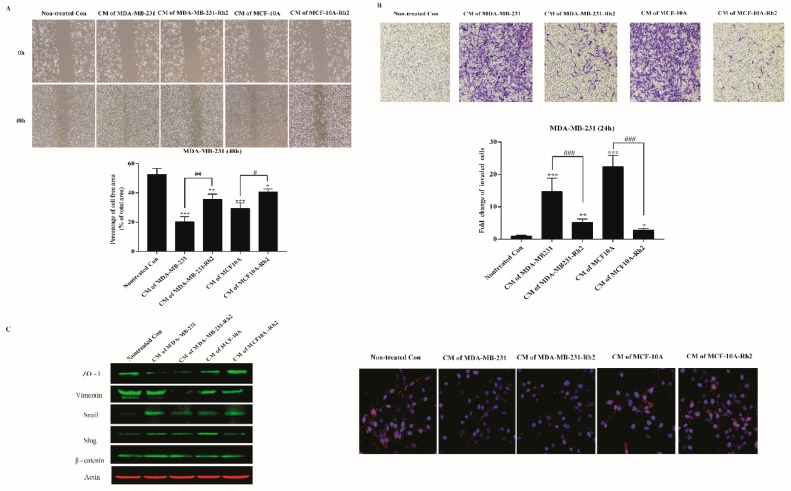
Rh2 inhibited SASPs-induced migration and invasion of breast cell lines. (**A**) MDA-MB-231 cells were cultured with conditioned medium from senescent MDA-MB-231 and MCF-10A cells treated with or without Rh2 for 48 h. The cell-free areas were imaged with microscope at 0 h and 48 h, respectively. The changes in cell-free area were calculated using CellProfiler Software (2.2.0). At least three wound scratches were analyzed per experiment. (**B**) Invasion of MDA-MB-231 cells (5 × 10^4^/well) as determined by 24-well plate transwell system. Cells on the lower side of membranes were stained and quantified. (**C**) Western blot analysis of representative epithelial–mesenchymal transition (EMT) markers of MDA-MB-231 cells after CM treatment. Immunostaining for the tight junction protein ZO-1(Red) and the nuclear regions were counterstained with DAPI (blue). * indicates *p* < 0.05 versus Nontreated con, ** indicates *p* < 0.01 versus Non-treated con; *** indicates *p* < 0.001 versus nontreated con. **^##^** indicates *p* < 0.01 versus CM alone group. **^###^** indicates *p* < 0.001 versus CM alone group.

To address the possibility that SASP (high secretions of IL-6 and IL-8) from senescent cells affects carcinoma cells migration, we examined the consequences of treatments with conditioned medium (CM) on the motogenic response of human breast cancers. Monolayers of MDA-MB-231 cells were scraped to create a cell-free area, and cell migrations were evaluated 48h later. Conditioned medium from senescent cells produced a marked increase in breast cancer migration (Figure 3A). As expected, quantitative assay showed that CM of MDA-MB-231 induced significant migration than that of non-treated con (*p* < 0.01). Importantly, similar to CM of MDA-MB-231, CM of MCF-10A also strongly stimulated breast cancer migration (*p* < 0.01). As expected, Rh2 treatment notably inhibited these elevated migrations. For invasion assay, CM of both senescent cell lines vigorously stimulated the cancer cell invasion by over 10-fold, which were noticeably mitigated by Rh2 treatment (Figure 3B). Since epithelial–mesenchymal transition (EMT) gives rise to caner invasion, we measured several hallmarks by western blot and immunofluorescence analysis. Intriguingly, CM of MDA-MB-231 elevated the expression levels of beta-catenin and snail while reduced the level of ZO-1 when in comparison to nontreated control cells. Rh2 exposure exerted decreased levels of beta-catenin and snail, while no changes of ZO-1 were noted. Importantly, Rh2 treatment powerfully abated the expression of vimentin though no noticeable increase was observed in CM treated cells. CM of MCF-10A induced higher expression level of slug, but lower than that of ZO-1 in comparison to normal cells; these alterations were ameliorated by Rh2 treatment (Figure 3C).

### 2.4. Ginsenoside Rh2 Does Not Exert Senolytic but Suppress the Paracrine Effects of Sasp in Human Breast Cell Lines

**Figure 4:**
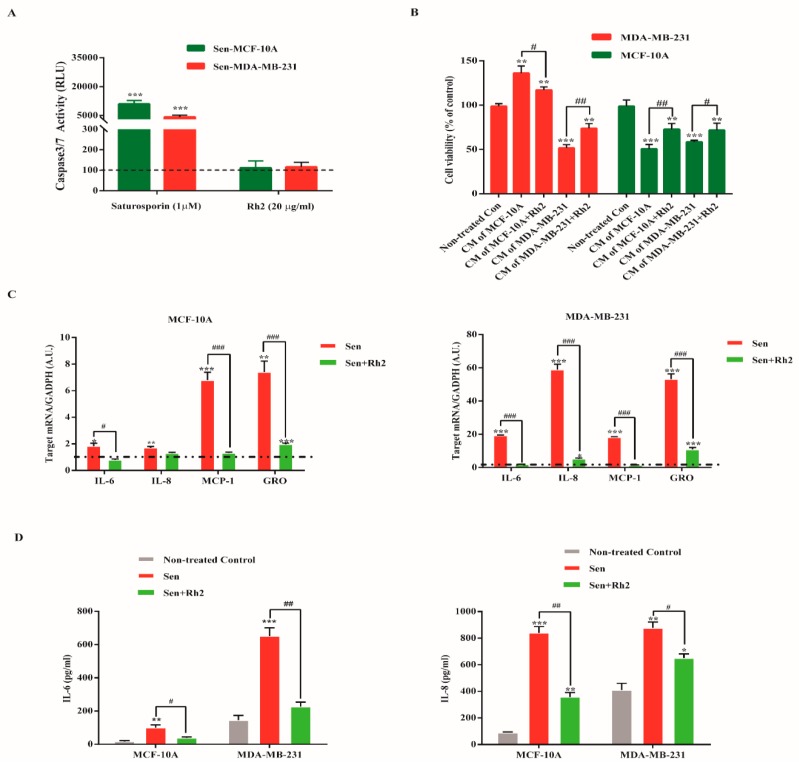
Rh2 suppressed paracrine effects of SASP in breast cell lines. (**A**) Senescent MDA-MB-231 and MCF-10A were exposed to Rh2 for 48 h, after which caspase 3/7 activity was quantified. The dotted line indicates fluorescence level in nontreated senescent cells. (**B**) MDA-MB-231 and MCF-10A cells were cultured with indicated CM for 24 h. The cell viability was assayed by WST-1 method. (**C**) Fold changes in mRNA levels of IL-8, IL-6, CXCL1, and MCP-1. The dotted line indicates expression level in control cells, set at 1 for each gene. *N* = 3. A.U., arbitrary units. (**D**) ELISA assay of IL-6 and IL-8. * indicates *p* < 0.05 versus Non-treated con. ** indicates *p* < 0.01 versus Non-treated con; *** indicates *p* < 0.001 versus Nontreated con. **^#^** indicates *p* < 0.05 versus CM group. **^##^** indicates *p* < 0.01 versus CM group. **^###^** indicates *p* < 0.001 versus CM alone group.

To check whether Rh2 exerts senolytic effects on both senescent cell lines, senescent MDA-MB-231 and MCF-10A cells were exposed to Rh2 (20 μg/mL) and caspase 3/7 activity was determined 48h later. Interestingly, Rh2 did not induce statistical apoptosis as compared to nontreated control group. Additionally, senescent cells in both cell lines showed apoptosis resistance as elaborated by reduced caspase 3/7 activity with staurosporin (Figure 4A).

Given senescent cells can reprogram neighboring cells through SASP, we determined whether ginsenoside Rh2 mitigated the cancer-promoting effect on MDA-MB-231 cells and senescent paracrine effect on MCF-10A cells. As seen in Figure 4B, CM from senescent MDA-MB-231 cells significantly inhibited the cell proliferation of MCF-10A cells, which was clearly ameliorated by treatment of senescent MDA-MB-231 cells with Rh2. On the contrary, CM from senescent MCF-10A cells exerted profoundly tumorigenic effect on MDA-MB-231 cells, while CM prepared from senescent cells treated with Rh2 attenuated the stimulated effect. Intriguingly, CM from both cell lines showed obviously self-senescence paracrine effect with manifested growth arrest. Additionally, CM from Rh2 treated senescent cells slightly rescued the cell proliferative ability.

Further, we measured the effect of Rh2 on the expression of some representative SASP genes associated with inflammation, proliferation, and invasion. As shown in Figure 4C, Rh2 significantly reduced the mRNA level of MCP-1 and CXCL1 in both senescent cells lines, while it selective suppressed that of IL-6 and IL-8. Additionally, the enzyme-linked immunosorbent assay (ELISA) further confirmed the highly secreted SASP components IL-6 and IL-8, which were significantly diminished by Rh2 treatment (Figure 4D).

### 2.5. Ginsenoside Rh2 Suppresses Potential Signaling Pathways Inferred SASP Secretion in Human Breast Cell Lines

**Figure 5:**
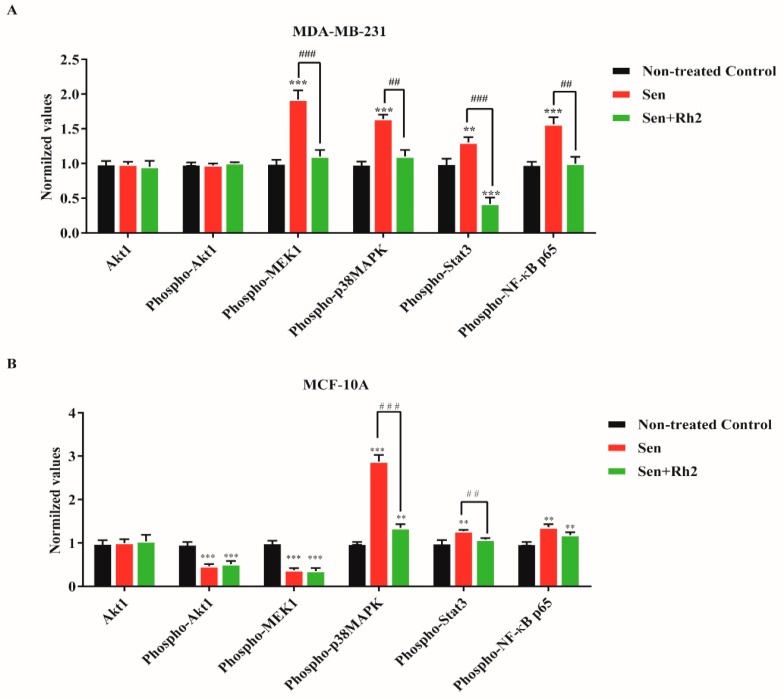
Rh2 suppressed potential signaling pathways regulating SASP in breast cell lines. Senescent MDA-MB-231 (**A**) and MCF-10A (**B**) cells treated with or without Rh2 were lysed and subjected to the Signaling Nodes Multitarget Sandwich ELISA assay. ** indicates *p* < 0.01 versus nontreated con; *** indicates *p* < 0.001 versus nontreated con. **^##^** indicates *p* < 0.01 versus CM alone group. **^###^** indicates *p* < 0.001 versus CM alone group.

To address the potential signaling pathways that activated SASP, we investigated the major pathways that regulate cell growth, differentiation, response to proinflammatory cytokines, growth factor receptors, inflammation, and cellular stress in the Signaling Nodes Multitarget Sandwich ELISA Kit. Endogenous levels of Akt1, phosphor-Akt(Ser473), phosphor-MEK1(Ser217/221), phosphor-p38 MAPK(Thr180/Tyr182), phosphor-Stat3(Tyr705), and phosphor-NF-κB p65(Ser536) were determined. For MDA-MB-231 cells, senescence significantly induced the phosphorylated forms of MEK1, p38, Stat3, and NF-κB p65, while no phosphorylation of Akt1 was noted. Treatment with ginsenoside Rh2 strongly reduced those elevations to the level of nontreated control but with further statistical decrease of Stat3 (Figure 5A). For MCF-10A cells, senescence evidently induced the phosphorylated forms of p38, Stat3, and NF-κB p65, while profoundly decreased phosphorylation of Akt1 and MEK1 was noted. Treatment with ginsenoside Rh2 only significantly reduced the elevated levels of phosphorylated p38 and Stat3, with no effects on Akt1, MEK1, and NF-κB p65 (Figure 5B). Interestingly, Rh2 exerted different suppression manner between two cell lines.

**Figure 6:**
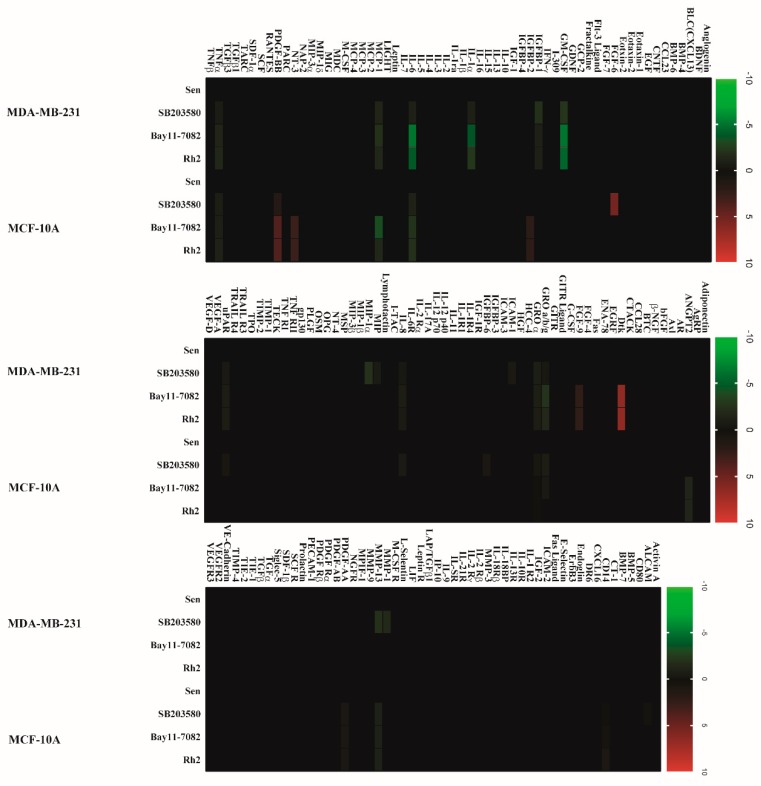
Rh2 suppressed SASP secretion in breast cell lines. Conditioned medium, prepared from senescent cells treated with DMSO, SB203580 (10 μM), Bay11-7082 (10 μM), and Rh2 (20 μg/mL), was analyzed with human cytokine antibody arrays. Levels of each cytokine factor in untreated cells were arbitrary set to zero. Data shown represent log2-fold change in expression relative to untreated cells. Signals higher than the senescent control are shown in red; signals lower than the senescent control are shown in green.

Given that two signaling pathways are well-documented regulator of SASP, we herein again verified that low-dose exposure to doxorubicin induced SASP with robust activation of p38 MAPK and NF-κB pathways. Additionally, inclusion of the p38 MAPK inhibitor SB230580 (10 μM), NF-κB inhibitor Bay 11-7082 (10 μM), or Rh2 (20 μg) in the incubation medium evidently suppresses the SASP in both cell lines (Figure 6).

## 3. Discussion

Cellular senescence, originally described as an irreversibly proliferative arrest in normal fibroblasts after a limited number of divisions [22], is an important tumor-suppressive mechanism. However, accumulated evidence challenged that cellular senescence exerts several deleterious biological functions encompassing tumorigenesis and age-related pathologies especially involving SASP [23]. Doxorubicin is the mainstay drug in the treatment for breast cancer. Intriguingly, since the required dose of chemotherapy drug to induce cellular senescence is much lower than that necessary to kill cells, breast cancer cells exposed to doxorubicin undergo widespread senescence [24]. In this study, we provided further evidence for the reciprocal effects of senescent breast tumor and normal cells under stimulation with clinical dose of doxorubicin. Additionally, we proposed that ginsenoside Rh2 is a potential candidate for the extenuation of chemotherapy-induced senescence bystander effect.

Doxorubicin induces the formation of DNA-Dox-topoisomerase II cleavable complexes and subsequent DNA damage, by which cellular senescence was initiated in many cancer cells. Clinically, the reported steady-state plasma level of doxorubicin achieved in patients receiving a total dose of 165 kg/m^2^ was 0.1 μM to avoid cardiac toxicity [25]. Accordingly, our results showed that 0.1 μM of doxorubicin successfully induced cellular senescence of breast carcinomas cell MDA-MB-231 and normal cell MCF-10A, as elaborated by typical flatten morphological changes, increased mitochondrial biomass, SA-β-gal expression, and redistribution of lysosomes. Additionally, elevated levels of senescence markers p16 and p21 were noted. Stable nuclear foci 53BP1 was observed, indicating the potential SASP development. Intriguingly, pretreatment or post-treatment of Rh2 does not affect the alterations of the senescence markers, suggesting Rh2 may acts downstream of signaling pathways involved in senescence.

To test the possibility that senescent cells develop SASP after challenge with doxorubicin, we assessed the levels of 120 secreted cytokines from CM using antibody arrays. As acknowledged, SASP presents variations relying on tissue and stimulus. A large number of cytokines consisting of SASP components secreted at significant level after doxorubicin induction, encompassing proinflammatory cytokines (e.g., IL-1α/β, IL-6, and IL-8), growth factor (e.g., PDGF and GM-CSF), chemokines (e.g., CXCL1 and MCP-1), and matrix remodeling enzymes (e.g., MMPs) were noted in the present study. Among the above typical SASP components, IL-1, IL-6, and IL-8 are reported to exert pleiotropic effects including maintenance of senescence, promotion of tumorigenesis and chemotherapy resistance [26]. GM-CSF has been reported to be overexpressed in a variety of human cancers including melanoma and hepatocellular carcinoma and was proposed to regulate tumor progression [27]. MCP-1 and CXCL1 (GRO) influence breast carcinogenesis by facilitating tumor growth and metastatic spread [28]. MMPs are another major SASP factor usually secreted by senescent fibroblasts, which can enhance the invasion of multiple epithelial cell types [29]. Apart from abovementioned SASP factors, certain cytokines were detected at remarkable levels as follows: insulin-like growth factor-binding proteins (IGFBPs), urokinase plasminogen activator (uPA), and fibroblast growth factor (FGF-6). These cytokines are closely associated with cancer invasion and metastasis [30]. Importantly, Rh2 treatment reversed these alterations of secreted cytokines in the present study. Intriguingly, nontumorigenic MCF-10A cell exhibited different pattern of SASP, with significant decreased secretion of PDGF-AA and PDGF-BB compared to nontreated normal cells, which might indirectly lead to tissue dysfunction and impaired regeneration. Similarly, Rh2 exposure restored the levels of PDGF-AA and PDGF-BB. Next, we measured the ability of SASP from both senescent cell lines to stimulate migration and invasion of MDA-MB-231 cells by a transwell system. Accordingly, significant migration and invasion were observed with CM supplement in culture medium, which were clearly suppressed by Rh2 treatment. Rh2 profoundly reduced the expression of EMT markers (beta catenin, slug, and snail) in MDA-MB-231 cells. Moreover, CM from Rh2 treated cells reduced carcinomas cell proliferation and restored normal cell doubling population when compared with CM-treated cells. Collectively, our results herein demonstrated that SASP is able to promote cancer progression and inhibit proliferation ability of normal cell in breast tissue, while Rh2 is a potential candidate for the amelioration of this deleterious effect. However, the present study did not explicitly address how Rh2 inhibited SASP-associated migration and invasion of malignant breast cancer cells. Chemotherapy-induced senescence has been proposed as a potential approach to combat cancer through induction of a persistent growth arrest state, whereas SASP is challenging, this possible strategy with reprogrammed and complicated tumor microenvironment as well as declined immune response in aging population.

How might Rh2 suppress SASP in senescent breast cell lines? Initially, DNA damage response and downstream activation of ATM are sufficient to activate certain SASP [31], which apparently can be induced by anticancer drug doxorubicin in the present study. Correspondingly, we detected DNA-SCARS via identifying 53BP1 foci in senescent cells. Currently, the NF-κB signaling pathway is proposed as the potential inducer and signaling pathway that activate NF-κB signaling subsequently triggers SASP [32]. Furthermore, the p38MAPK signaling pathway is a crucial inducer for SASP without genotoxic stress in human fibroblasts [33]. Consistent with these well-documented inducers, our results again confirmed the evident activation of p38MAPK and NF-κB signaling pathways in senescent breast cells. Additionally, we also verified noteworthy upregulation of Akt and MEK pathways, which are potential mediators for SASP development and maintenance. However, decreased expression of Akt and MEK pathway were noted in nontumorigenic cell MCF-10A. Concomitantly, inhibitors SB203580 for p38MAPK and BAY11-7082 for NF-κB significantly suppressed the SASP from both tumorigenic and nontumorigenic cells evidenced by antibody array test. Moreover, notable activation of Stat3 may potentiate the deleterious effect of doxorubicin-induced senescence, since this signaling pathway mediates immune suppression in tumor [34]. Impressively, for doxorubicin-induced senescent breast tumor cell line MDA-MB-231, Rh2 not only suppressed the accepted p38MAPK and NF-κB pathways but also prohibited Stat3 pathway. Together, Rh2 may attenuate SASP development through regulation of above mentioned multipathways.

## 4. Materials and Methods

### 4.1. Cell Culture

Human breast cancer cells MDA-MB-231 and normal breast cells MCF-10A were purchased from the American Type Culture Collection (ATCC). Breast cancer cell line MDA-MB-231 was maintained in high glucose Dulbecco’s Modified Eagle’s Medium (Themro Fisher Scientific, Seoul, South Korea), supplemented with 10% Fetal Bovine Serum and penicillin/streptomycin (Invitrogen, Seoul, South Korea). MCF-10A was maintained in Dulbecco’s Modified Eagle’s Medium/F-12 (Thermo Fisher Scientific, Seoul, South Korea), supplemented with Mammary Epithelial Cell Growth Medium SingleQuot Kit Supplement & Growth Factors (Lonza, CC-4316, Alpharetta, GA, USA) and 100 ng/mL cholera toxin (Sigma, St. Louis, MO, USA). The cells were incubated at 37 °C in a 5 % CO_2_ atmosphere.

### 4.2. Reagents

Ginsenosides Rg1, Re, F1, Rh1, Rh1, PPT, Rb1, Rd, Gyp75, F2, Rg3, Rh2, CK, and PPD, with a purity of more than 98%, were prepared with High Performance Liquid Chromatography (HPLC, Agilent, Seoul, South Korea). Each ginsenoside was dissolved in dimethyl sulfoxide (DMSO, St. Louis, MO, USA) as 10 mg/mL solution. Bay 11-7082, SB203580 and doxorubicin were purchased from Sigma (St. Louis, MO, USA).

### 4.3. Senescence Induction and Assessment

Human breast cancer cells MDA-MB-231 and normal breast cells MCF-10A were induced to senescence by exposure to doxorubicin in complete culture medium. Briefly, proliferating cells were treated with indicated concentrations of doxorubicin for 72 h. Later, cells were scored for senescence markers, including senescence-associated β-galactosidase (SA-β-gal) activity and the amount of persistent DNA damage foci. SA-β-gal staining was performed using a SA-β-gal kit (#9860, Cell Signaling Technology Inc., Beverly, MA, USA) in accordance with the manufacture’s manual. The cells were fixed for 15 min at room temperature, then rinsed with PBS and stained with staining solution at a final pH of 6.0 for overnight (at least 16 h). The SA-β-gal positive cells develop blue color and were counted under a phase-contrast microscope. DNA damage foci were estimated by immunostaining for 53BP1. For DNA damage foci and SA-β-gal positivity, random fields were selected. Fluorescent images were quantified using CellProfiler (2.2.0, Cambridge, MA, USA), an open source software program. SA-β-gal staining was quantified by researcher that was blind to the treatments.

For SASP development, cells were treated with 100 nM doxorubicin for 2 cycles (day 0 and day 4) for 10 days. Specific inhibitor of p38MAPK (SB203580), NF-κB inhibitor, Bay 11-7082, and ginsenoside Rh2 were added at day 6. Then 48 h later (day 8), cultures were replaced with fresh serum-free medium. Lastly, medium were collected and prepared for the subsequent tests (day 10).

### 4.4. Cell Proliferation Assay

Cell viability (proliferation) was evaluated by the WST-1 assay, which is based on the enzymatic cleavage of the tetrazolium salt WST-1 to formazan by cellular mitochondrial dehydrogenase present in viable cells. In brief, after 72 h treatment, 20 μL of WST-1 was added to each well and the plates were incubated at 37 °C for 2 h. The plates were then centrifuged and 100 μL of the medium was withdrawn for measuring the absorbance value at a wavelength of 450 nm using a microplate reader (Tecan, Männedorf, Switzerland).

Seventy-two hours after senescence induction, cells were also assessed by immunostaining for Ki-67, a key proliferation marker. Fluorescent images were captured by a Nikon i2 U microscope (Tokyo, Japan).

### 4.5. Assay of Caspase 3/7 Activation

Cells were plated in 12-well plates and subjected to senescence induction. Six days after that, DMSO or ginsenoside Rh2 was added into culture medium. Forty-eight hours after addition, live imaging was initiated by 30 min preincubation of CellEvent Caspase 3/7 green detection reagent (5 μM, Thermo Fisher Scientific, Seoul, South Korea) using a Nikon i2 U microscope (Tokyo, Japan) and quantification was measured by microplate reader (Tecan, Männedorf, Switzerland).

### 4.6. Cytokine Antibody Array

The conditioned medium (CM) for antibody analyses were prepared by washing approximately 6 × 10^6^ presenescent and senescent cells 3 times with PBS, and incubating them with serum-free medium for 48 h. The conditioned medium were collected and the remained cells were counted to normalize conditioned medium volumes for cell number. Then medium were centrifuged for 20 min at 5000 rpm, filtered through 0.22 μM bottle-top filters (Sartorius Stedim Biotech, Göttingen, Germany) diluted with serum-free medium to a concentration equivalent to 1 × 10^6^ cells per 1.5 mL, and applied to antibody array (Ray Biotech, Norcross, GA, USA). The signals were detected with Odyssey-LC chemiluminescent imaging system. Signals were averaged and expressed as described in the figure legend.

### 4.7. Migration and Invasion Assay

MDA-MB-231 and MCF-10A cells were plated in 24-well plates (5 × 10^4^ cells per well) in a complete corresponded medium. Cells were scraped off from the bottom of a culture plate using a pipette tip to produce a cell-free area. Cells were washed with DMEM or DMEM: F12 (1:1) to remove the cell debris and incubated with indicated conditioned media in 3% FBS prepared from senescent MDA-MB-231 and MCF-10A cells treated with or without ginsenoside Rh2 for 24 h. The wound areas were captured at 0 h and 48 h and quantified using CellProfiler Software (2.2.0, Cambridge, MA, USA).

MDA-MB-231 cells were serum-starved overnight, trypsinized, then seeded in the upper chamber with Matrigel-coated transwells in serum-free medium, with cells migrating towards the lower chamber in response to SASP-containing CM (R&D systems, Minneapolis, MN, USA). Cells on the lower side of the membranes were stained with 0.1% crystal violet (Sigma) after 24 h and enumerated.

### 4.8. Quantification of SASP Major Factors

IL-6 and IL-8 levels in conditioned media were quantified using Human IL-6 and IL-8 ELISA Ray Biotech protocol respectively.

Major SASP factors were analyzed by real-time PCR. Total RNA was prepared with the RNeasy Micro Kit (Qiagen, Germantown, MD, USA). qRT-PCR reactions were performed using the QuantiNova SYBR Green RT-PCR Kit (Qiagen) according to the manufacture’s protocol. Primer/probe sets for human IL-6, IL-8, MCP-1, GRO were used: IL6F: 5′-GCCCAGCTATGAACTCCTTCT-3′; IL6R: 5′-GAAGGCAGCAGGCAACAC-3′; IL-8F: 5′-AGACAGCAGAGCACACAAGC-3′; IL-8R: 5′-ATGGTTCCTTCCGGTGGT-3′; MCP-1F: 5′-AGTTCTTGCCGCCCTTCT-3′; MCP-1R: 5′-GTGACTGGGGCATTGATTG-3′; CXCL-1F: 5′-TCCTGCATCCCCCATAGTTA-3′; CXCL-1F: 5′-TCCTGCATCCCCCATAGTTA-3′; CXCL-1R: 5′-CTTCAGGAACAGCCACCAGT-3′.

The Ct-value for targets and endogenous control (GADPH) were used to calculate the relative expression of the gene of interest. Samples were determined in triplicate.

### 4.9. PathScan^®^ Signaling Nodes Multitarget Sandwich ELISA Assay

Presenescent and senescent MDA-MB-231 and MCF-10A cells were collected after SASP was fully developed, and then lysed with ELISA lysis buffer. Cell lysates were applied to specific assay formulations for the indicated target proteins according to the manufacture’s protocol (Ray Biotech, Norcross, GA, USA). Briefly, microwell strips were unsealed and placed at room temperature. One-hundred microliters of sample diluted with equal amount of sample diluent were applied to well and incubated overnight at 4 °C. Then the microwell strips were washed with buffer and incubated with detection antibody for 1h at 37 °C, followed by HRP-linked secondary antibody for 30 min at 37 °C. Next, TMB substrate was added and incubated for 10 min at 37 °C. After adding stop solution to each well, the microwell strips were measured using a microplate reader (Spark 10M, Tecan, Männedorf, Switzerland) at a wavelength of 450 nm within 30 min.

### 4.10. Western Blotting

Cell lysates were collected and prepared from indicated treatments. Lysates were subjected to 10% SDS-PAGE gels; separated proteins were transferred to polyvinylidene difluoride membrane for 1 h at 110 V. Membranes were blocked and incubated overnight at 4 °C with the following primary antibodies: p16, p21, ZO-1, Vimentin, Snail, Slug, and beta-catenin (rabbit monoclonal, 1:1000, CST, USA), with beta-actin (mouse monoclonal, 1:2000, CST, Norcross, GA, USA) as loading control. Membranes were washed and incubated with horseradish peroxidase-conjugated (1:5000; CST, Norcross, GA, USA) or IRDye 800CW or IRDye 680RD (1:10000; Li-COR, Lincoln, NE, USA) for 1 h at room temperature and washed again. Signals were detected by Odyssey-Fc imaging system (Image Studio, ver5.2, Li-COR, Lincoln, NE, USA).

### 4.11. Fluorescence Microscopy

Cells with various indicated treatments were washed twice with PBS, then cells were fixed with 4% paraformaldehyde for 10 min and permeabilized with 0.15% Triton X-100 in PBS for 15 min at room temperatures. Cells were then blocked with 3% BSA for 30 min and incubated with indicated primary antibody against ZO-1 (rabbit monoclonal, 1:2000, CST, USA) overnight at 4 °C, followed by incubation with Alexa fluorescein-labeled secondary antibodies (1:200, Thermo Fisher Scientific, Seoul, South Korea) for 1h and mounted with DAPI (Thermo Fisher Scientific, Seoul, South Korea). Images were captured with a Nikon i2 U microscope (Japan).

### 4.12. Statistical Analyses

All data that show error bars are presented as mean ±s.e.m. The significance of difference in the mean was determined using Student’s *t*-test and one-way Analysis of Variance (ANOVA) unless otherwise mentioned. *p* < 0.05 was considered significant. All calculations were performed using GraphPad Prism software (7.0, San Diego, CA, USA).

## 5. Conclusions

In summary, our results provided Rh2 as a potential candidate for ameliorating chemotherapy-induced senescence bystander effect, which might be associated with such age-related pathology breast tumor progresses and tissue damage.
